# Implanted cortical neuroprosthetics for speech and movement restoration

**DOI:** 10.1007/s00415-024-12604-w

**Published:** 2024-10-24

**Authors:** William R. Muirhead, Hugo Layard Horsfall, Christine Aicardi, Jacques Carolan, Harith Akram, Anne Vanhoestenberghe, Andreas T. Schaefer, Hani J. Marcus

**Affiliations:** 1 The National Hospital for Neurology and Neurosurgery, University College London Hospitals NHS Foundation Trust, London, UK; 2 The Francis Crick Institute, London, UK; 3 UCL Queen Square Institute of Neurology, University College London, London, UK; 4 Faculty of Natural, Mathematical & Engineering Sciences, King’s College London, London, UK; 5 Wolfson Institute for Biomedical Research, University College London, London, UK; 6 School of Biomedical Engineering and Imaging Sciences, King’s College London, London, UK

**Keywords:** Neurotechnology, Brain–computer interface, Neuroprosthetic, Neuromotor prosthesis, Motor neuroprosthesis, Neurological disease

## Abstract

Implanted cortical neuroprosthetics (ICNs) are medical devices developed to replace dysfunctional neural pathways by creating information exchange between the brain and a digital system which can facilitate interaction with the external world. Over the last decade, researchers have explored the application of ICNs for diverse conditions including blindness, aphasia, and paralysis. Both transcranial and endovascular approaches have been used to record neural activity in humans, and in a laboratory setting, high-performance decoding of the signals associated with speech intention has been demonstrated. Particular progress towards a device which can move into clinical practice has been made with ICNs focussed on the restoration of speech and movement. This article provides an overview of contemporary ICNs for speech and movement restoration, their mechanisms of action and the unique ethical challenges raised by the field.

## Introduction to implanted cortical neuroprosthetics and aims of the review

Implanted cortical neuroprosthetics are a novel class of active implantable medical devices which may support improve the quality of life for patients with a diverse range of conditions including paralysis, anarthria and blindness [1, 2]. Over the past two decades, the field has undergone significant progress with very high device performance now possible within a laboratory setting. The coming decades are likely to see an increase in the number of these devices entering clinical trials and perhaps even transitioning into routine clinical practice.

An implanted cortical neuroprosthetic (ICN) is surgically *implanted* (distinguishing it from a non-invasive, for example, EEG-based system) [3], directly records from or stimulates the *cortex* of the brain (unlike, for example, cochlear implants) [4], and is *prosthetic* insofar as it aims to replace a neural pathway rather than simply modulating it (this separates them from, for example, deep brain stimulation platforms which primarily modulate a dysfunctional circuit rather than bypassing it) [5]. The term ‘brain–computer interface’ is sometimes used to refer to ICNs but the definition of ‘brain–computer interface’ is inconsistent in the literature and we avoid using it here for this reason [3–5]. Implanted systems are necessarily invasive; compared with non-implanted systems, this typically increases the risk but has the advantage of increased spatiotemporal resolution [6]. At present, the highest performance neuroprosthetics are implantable and they are the focus of this article [7].

The principal applications of ICNs in clinical studies have been for people with impairments of vision [8], speech [9], and movement [10]. Virtual movement can facilitate digital device control as well as communication (through for example a virtual keyboard) and people with both paralysis and anarthria have been identified as a candidate group for ICNs which facilitate communication either through direct speech synthesis or virtual device control [9, 11, 12]. For patients with severe communication impairments, even basic implants that enable the users to engage with assistive technology can have a significant impact on their quality of life. [13]

This review provides an overview of contemporary ICNs for speech and movement restoration, describes their potential clinical benefits and presents some of the ethical considerations raised by this field.

## Interpreting brain activity: clinical benefits of decoding intention

### The clinical benefits of decoding speech and movement intention

The terms *neuromotor prosthetic* or more recently *motor neuroprosthetic* is often used to describe an ICN which records a signal from motor cortex and translates that into movement (frequently virtual movement of a cursor on a screen) [14–16]. In the context of the motor signal being used to control a digital device, this output has been described as a *digital motor output* [16].

**Fig. 1:**
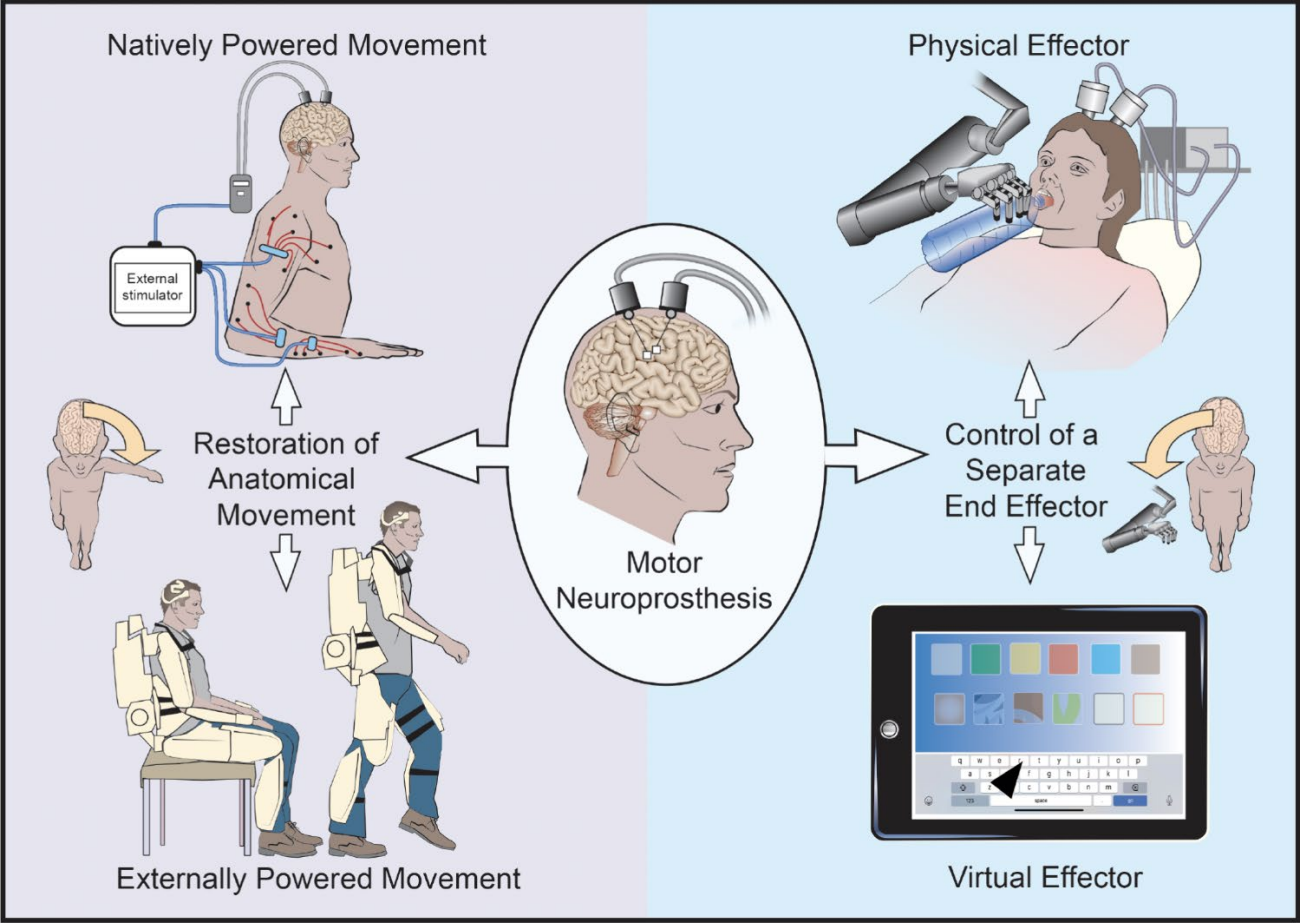
Categories of movement restoration and effector control that can be driven by a motor neuroprosthesis

For someone with paralysis, a motor neuroprosthetic can be used either to restore their own anatomical movement, or to provide control of an external effector (Fig. 1).

Restoration of anatomical movement has been achieved through activating the user’s muscles by stimulating the spinal epidural space or muscles directly [10, 17]. An alternative approach is to power the user’s movement using an external orthosis. In this application, the neuroprosthetic controls movement of the orthosis and the powered orthosis moves the body of the user. This orthosis could be a rigid exoskeleton or based on soft robotic principles [18, 19].

An alternative approach is to provide direct control over a separate external end effector. Effector control can be *virtual*, for example, allowing the user to control a mouse to operate a graphical user interface on a tablet computer [12]. Control can also be over a *physical* effector, such as a table-mounted robotic arm, which could support the user with activities of daily living, or an electric wheelchair [20].

An ICN can also contribute to rehabilitation. This is particularly relevant for motor neuroprostheses which are targeted towards restoration of limb movement. There are two mechanisms by which this could occur. First, by reconnecting intention and action, it is hypothesised that a neuroprosthetic can induce neuroplastic changes in native circuits. Second, by facilitating movement in disused muscle groups thus preventing them from becoming deconditioned. This can reduce the likelihood of contractures and permanent deformity, as well as bone resorption and osteopenia from reduced weight bearing stress on long bones [21, 22]. The use of ICNs to facilitate recovery through a combination of immediate movement restoration and rehabilitation has already been demonstrated, and may become the predominant paradigm through which these devices enter clinical use [21].

Restoration of communication is frequently the aim of an ICN. Providing control over an electronic device with virtual movement has been used to facilitate virtual typing with the user able to move a computer cursor across a virtual keyboard [23]. An alternative approach that has been used has been to use the motor signal from the neuroprosthetic to provide a mouse ‘click’ which can be combined with an eye-tracking gaze control interface. In this way, the user is able to move across options with their eye movement and select an option from a graphical user interface using the click provided by the ICN [15].

Whilst control over an external device can facilitate communication, there have also been ICNs designed to directly decode neural signals into speech by recording from the face and laryngeal region of the motor cortex [7]. Directly decoding these signals into speech allows for a more seamless and intuitive communication experience for the participant. Incorporation of recordings of a participant's voice from prior to their injury as well as the use of a virtual avatar modelled on their facial expressions has allowed for the speech to be virtually embodied with some of the sound and appearance of the participant themself [9].

### Approaches to recording neural activity

**Fig. 2:**
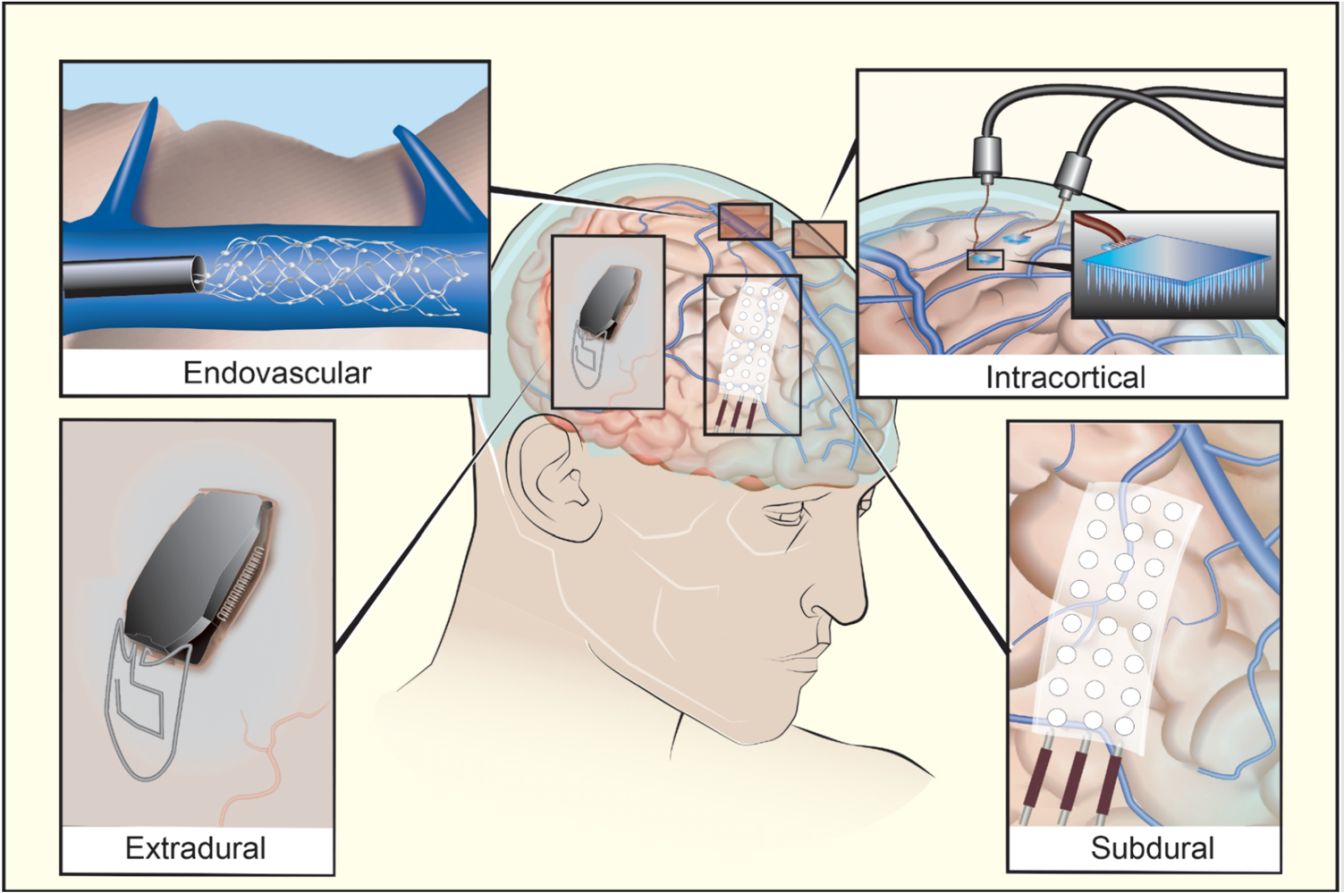
Illustration of devices in the four different anatomical compartments in which motor neuroprostheses have been implanted in human trials

Implanted cortical neuroprosthetics which have reached clinical trials have placed recording electrodes in one of four anatomical compartments: intracortical, subdural, extradural and intravascular (Fig. 2).

**Fig. 3:**
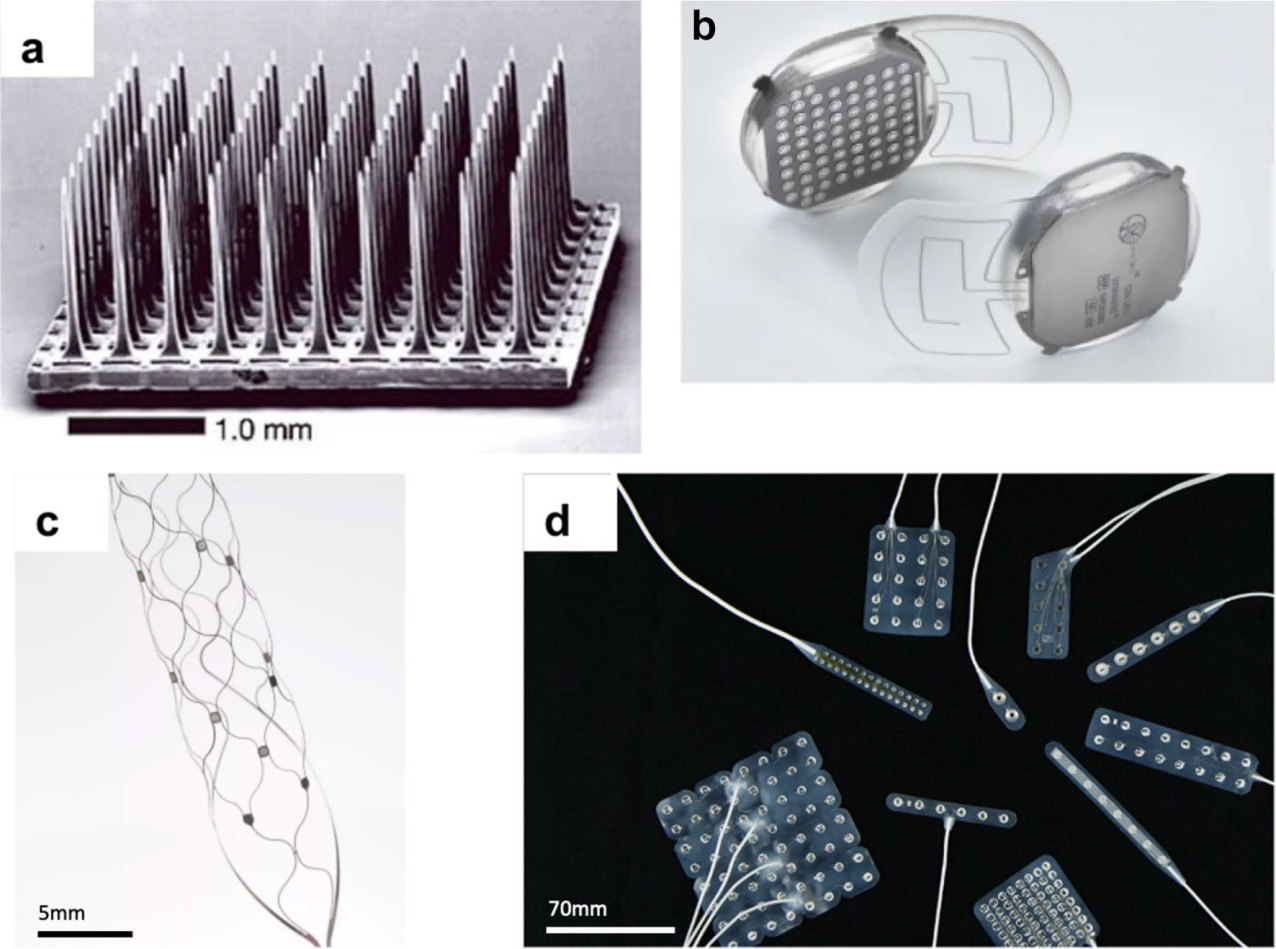
(**a**) A Utah Microelectrode Array (panel figure adapted from [14] used with permission) (**b**) WIMAGINE extradural electrode array (panel figure adapted from [27] used with permission); (**c**) Stentrode endovascular neuroprosthesis (panel figure from Synchron Corporation [28] used with permission); (**d**) Subdural electrode arrays (arrays in the panel figure are adapted from Ad-Tech Medical and used for seizure monitoring [29] used with permission) Figure adapted from sources referenced and images reproduced with permission

The greatest experience has been with intracortical microelectrode arrays (MEA). The most commonly used is the “Utah array” (Blackrock), a 4 mm square MEA with typically 100 1.5 mm silicon electrode shanks. These intracortical arrays are inserted into the brain surface to allow for dense local electrode coverage and multiple MEAs may be placed to increase spatial coverage of the brain. MEAs require transgression of the cortex itself although the electrodes themselves are relatively small—the shanks of a Utah array are 80 µm in diameter (Fig. 3a) [24]. More recently a system of flexible electrode “threads” has been developed (Neuralink [25]), compared to a Utah array, these threads have a much higher density of recording sites and the advantage of being able to move with the brain parenchyma. The 64 flexible threads on their first implant are 10–12 microns in diameter and each carry 16 electrodes. The first human implantation of a flexible electrode thread system was notable for retraction of some of these recording threads from the brain, which reduced the number of useful recording sites for the INC [26].

Subdural electrodes, arranged on an electrocorticography (ECoG) or micro-ECoG array, can be placed without transgressing the pia once the subdural space is accessed by craniotomy. They arguably offer the greatest potential for spatial coverage of all the implanted approaches [30] (Fig. 3d). A relative disadvantage of this approach, compared to an MEA for example, is that it typically requires access to a relatively large surface of neocortex to record signal from. Presently, this necessitates a large craniotomy although some device manufacturers are developing minimally invasive techniques to facilitate ECoG-based approaches [31].

Extradural electrodes do not require opening the dura, potentially reducing the risks of brain injury and cerebral infection, as the dura forms an anatomical barrier between the device and cortical tissue. By removing a disc of calvarial bone and using the ICN device itself as the cranioplasty, it is possible to place a relatively large implant with minimal soft tissue distortion. In this design, the electrodes can be integrated directly into this device so there is no relative movement between the electrodes and the rest of the implant (Fig. 3b) [32]. A disadvantage of this approach is that a layer of dura intervening between the recording electrode and the brain is likely to reduce the resolution compared with a comparable electrode in the subdural compartment.

The first intravascular neuroprosthetics have recorded from electrodes mounted on a stent in the sagittal sinus [15]. By deploying the stent adjacent to the precentral gyrus, it is possible to record correlates of movement intention principally from the motor leg area. An advantage of this approach is that electrodes can be deployed without the need for craniotomy, and the neurointerventional procedure of stent deployment into venous sinuses is well established [33]. As with extradural systems, a limitation of deployment within the sagittal sinus is that the neural signal recorded is attenuated by an intervening layer to the cortical surface—the dura of the sinus itself. A specific risk of intravascular stents is inducing venous sinus thrombosis, although in trials this has been preempted by antiplatelet prophylaxis with no thrombosis reported to date (Fig. 3c) [15].

### Signal decoding, calibration and training

Once a signal is recorded from the brain, a mapping function is needed to relate features of the neural signal to a movement or speech intention. This algorithm is called a *decoder*. The decoder is calibrated to the specific neural activity of an individual user by a training period, during which the user attempts (or imagines attempting) relevant tasks. The spatiotemporal relationship between the neural activity recorded and the intended action is the basis for calibration of the decoder.

Recent progress in ICNs has been made possible by advanced decoding strategies combining, for example, Bayesian classifiers, recurrent neural networks and language models for word prediction [7, 9]. The decoding strategy is also dependent upon whether the source signal comprises unit activity, local field potential or ECoG signal. To illustrate the principles involved in decoding, we present a relatively simple example of a decoder from one of the earliest clinical studies in intracranial neuroprosthetics—this is not intended to be representative of the mathematical complexity behind how the most advanced current decoders work (see, for example, Fig. 6), but this early example is an excellent illustration of the principles and highlights some of the challenges to these techniques transferring directly into a clinically useful device.

**Fig. 4:**
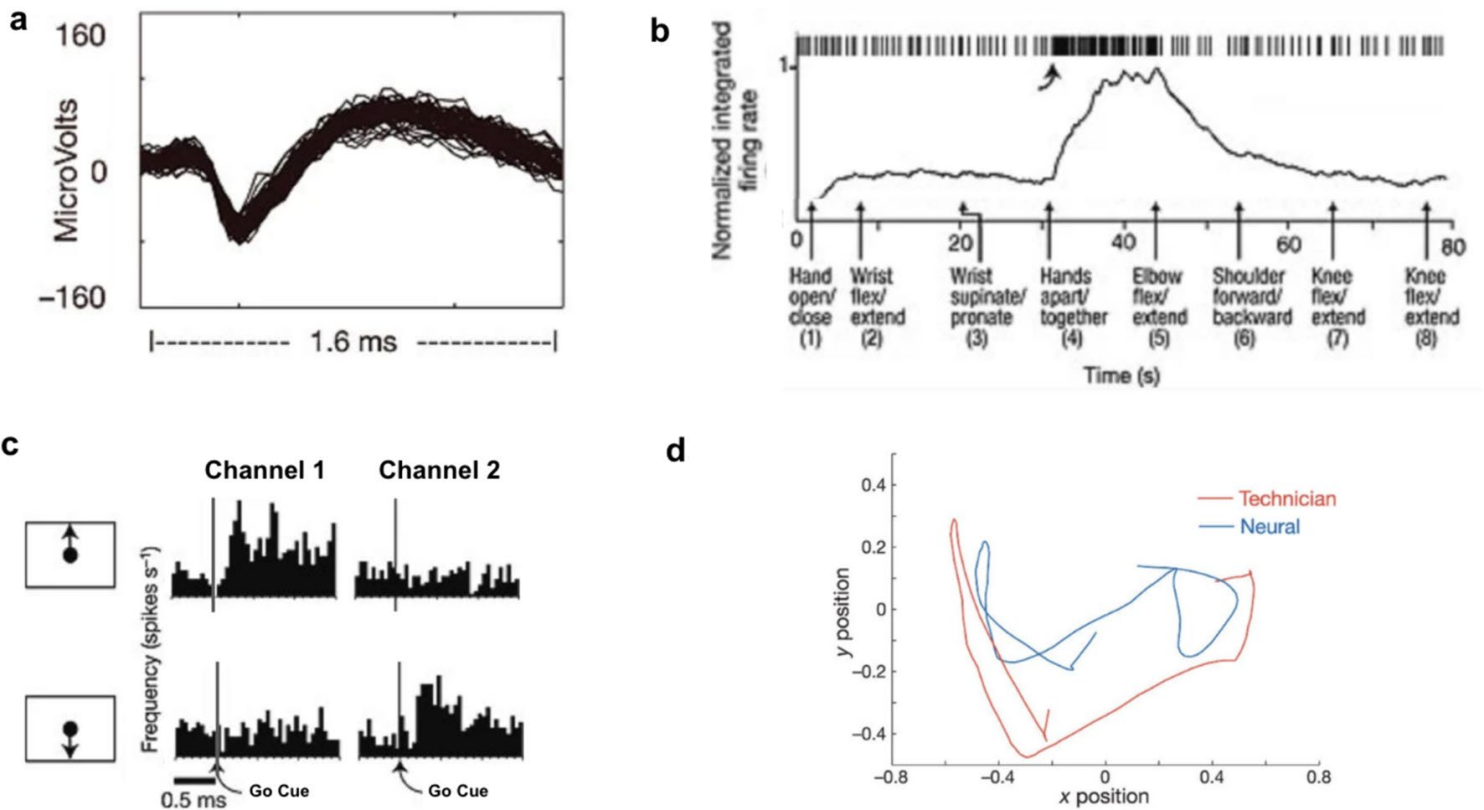
Neural recordings from the first human user of a microelectrode array based neuroprosthetic (**a**) A well-isolated single unit recording from a single electrode (trace shows the superposition of 80 waveforms); (**b**) Over 80 seconds the participant was asked to imagine performing a series of movements in the arm contralateral to the array. Spiking activity of a recorded unit is shown along the top of the panel with the normalised integrated firing rate immediately below that. This unit demonstrates an increased firing frequency with the instruction to move hands apart/together; (**c**) Spike rates for two units recorded simultaneously during the performance of movement of the on screen neural cursor. The unit recorded in channel 1 demonstrates increased firing with the cue to move the cursor upwards but not downwards. Conversely, the unit recorded in channel 2 demonstrates increased firing following the cue to move the cursor downwards but not upwards; (**d**) Research technician and participant neural cursor traces during a 5 second period of the participant tracking the cursor; Panel figures adapted from [14] and used with permission. Channel numbers altered for simplicity of presentation Figure adapted from source referenced and images reproduced with permission

The first human implantation of a Utah microelectrode array was into the hand area of the primary motor cortex (BrainGate trial) [14]. Electrical activity caused by the discharge of individual or small groups of neurons were recorded from electrodes in the array—these electrical discharges are termed *units* (Fig. 4a).

Changes in the unit activity recorded by each of these electrodes form the basis of the control signal. By asking the participant to imagine performing different movements, they are able to modulate this signal, in particular to increase the firing frequency of particular units (Fig. 4b).

For the participant to be able to control the movement of a cursor on a screen, a decoder was trained. By asking the participant to imagine physically moving a cursor on a screen in particular directions, units which have an increased firing frequency associated with this imagined movement can be identified. If a direction of movement is identified which is associated with an increased firing frequency for a particular neuron, then this neuron is said to have directional *tuning*.

To control the movement of a cursor on a screen in a two-dimensional space, a decoder was built by creating a mapping (often referred to in the literature as a “filter”) between the set of firing frequencies of the sampled units to a two-dimensional output signal. This mapping was initially constructed by asking the participant to imagine tracking a cursor moved by a member of the study team. Once an initial mapping between neural activity and intended cursor direction was established, it could then be used to place a neurally controlled cursor on the screen. Further calibration with the participant moving the neural cursor on screen enabled refinement of the mapping (closed-loop calibration).

In this first demonstration of a motor neuroprosthetic in the BrainGate trial, the user’s control of the neural cursor was limited to the context of research sessions. Each of these sessions would begin with the training of the decoder, i.e. even if the user wanted to perform the exact same task they had undertaken the day before they would still need to go through the calibration task to calibrate the decoder [34].

Retraining of the decoder was needed because of nonstationarities in the neural signal, i.e. the relationship between neural signal and movement intention is not stable over time. This is likely to be due in part to small movements of the array, changes in the local cellular environment, but also because of the changes in the tuning of the individual neurons themselves. This phenomenon, which is described as *representational drift*, [35] means that a highly directionally tuned neuron which has an increased firing rate associated with imagined leftwards movement one week may have no such tuning the following week. Consequently, without recalibration, the accuracy of a decoder declines over time.

To reduce the need for retraining, a strategy which has been subsequently employed is to continuously recalibrate the decoder based upon the data implicitly provided by the user as they use the interface [23]. Rather than relying upon a training epoch in which the user is asked to move a neural cursor towards a target specified by the research team, the intended target can be retrospectively inferred during active use and these data are used to calibrate the decoder. The advantage of this technique (*retrospective target inference*) is that it reduces the need for the user to disrupt their device use to undertake recalibration tasks.

Whilst the earliest implementations of microelectrode-based BCI required daily retraining sessions immediately prior to every use, in the most recent applications of continuous online recalibration, it has been possible to demonstrate stable decoding without retraining for more than 1 year of device use with over 90% accuracy in an online handwriting task [14, 36].

ECoG signals rather than unit activity can also be used as the control signal for a neuroprosthetic. Functional cortical activity is typically associated with an increase in high gamma power and a corresponding reduction in low-frequency power [37]. In a typical application, neural signals recorded by ECoG electrodes can be processed to extract both bands of high-frequency ‘high gamma’ activity (e.g. 70–150 Hz) as well as low-frequency signals (e.g. 0.3–17 Hz) [9, 38]. The power in these bands can then be used as the control signal for a neuroprosthetic. ECoG signals, representing the average activity of large numbers of neurons, might contain less information but have the advantage of increased stability, and consequently, do not require the same retraining as an MEA-based neuroprosthetic [39].

Whichever source signal is used, an important consideration for clinical translation is how much input is needed from engineers or neuroscientists when the system is in use. In applications based in university labs, participants are often engaged in training sessions and activities with an engineer or neuroscientist working alongside them on optimising device function. As more commercially ready devices are being developed for home use, there is a move towards engineers developing software that can run on a smartphone or tablet computer and enable the user to undertake device training themselves without the need for direct supervision from an engineer or researcher. In this paradigm, industry will manufacture devices with paired software that can be prescribed by a treating clinician and trained by the user in their own home.

### Challenges and considerations for long-term implantation

High-performance intracranial neuroprosthetics for both speech synthesis and device control have been demonstrated in research settings [7, 12]. This performance has yet to transfer into a reliable device that is suitable for the participant to use any time at home as their primary means of communication. There are two challenges that are specific to these cortical neuroprosthetics when compared with intracranial devices that have moved into routine practice such as cochlear implants or DBS.

As outlined above, one key challenge is signal stability. Most of the experience using implanted cortical neuroprosthetics has been with MEA where there is considerable change in the neural control signal over time. Some of these changes—hypothesised to be due both to small movements of the probe within the brain as well as due to representational drift—can be overcome with recalibration of the decoder. However, recalibration of the decoder has typically relied upon regular training sessions with highly motivated cognitively preserved participants. The intensive input needed from both the research team and the participant has meant that recruiting sites are typically limited to very small numbers of participants, often just one. The need for retraining has been a significant barrier to the development of a device which the participant can use at home without the supervision of the research team.

Another changes seen with MEA is a decay of the quality of the neural signals which starts within months after implantation [40, 41]. This is thought to be a consequence of both degradation of the arrays and a biological changes in the implanted tissue, including glial encapsulation and neuronal loss surrounding the arrays [42, 43]. To overcome this, many innovative electrode designs are being produced using new materials, for which we lack long-term stability data. Multi-layered structures can delaminate, metals can corrode and even silicon passivation layers are known to hydrate after long immersion periods. More studies are needed to understand their degradation mechanisms in the body environment and whether by-products of this degradation can alter their biocompatibility [44, 45].

The second challenge is data transfer. Neuromodulatory systems, like DBS, are typically programmed wirelessly. Once stimulation settings are determined, the device runs without requirement for data transfer between the implanted device and external system. Cochlear implants do require data transfer but the amount of data transferred is much lower than can be generated using, for example, a microelectrode array.

A typical goal of neuroprosthetics for speech or motor restoration is transmission of data (from which to infer intention or control commands) to an external effector. Intracortical neuroprosthetics typically require high sampling rates (often several kHz for unit recordings) and consequently generate significant volumes of data. Electromagnetic transfer of data using fully implanted systems is limited due to factors such as signal attenuation from passing through the tissues, heating of the device, and challenges in optimising transmission with sufficiently small implanted devices [46, 47]. Performing data preprocessing on the internalised part of the system can reduce the amount of data that needs to be transmitted, but this introduces power consumption and heat dissipation challenges. Consequently, fully implantable neuromodulatory systems have typically relied on ECoG signal with a lower demands for data transmission [15, 38].

## Landmark implanted cortical neuroprosthetic studies in humans

The first report of an ICN was in 1998 using a *neurotrophic electrode*. The study participant was ventilator-dependent with severe amyotrophic lateral sclerosis (ALS) and communicated using eye-movements. Two glass and gold electrodes with neurotrophic factors were implanted into the hand area of the motor cortex. The user was able to actively modulate the firing rate of units recorded by one of the electrodes but further testing was limited by intensive care admissions [48].

The first multicentre implanted cortical neuroprosthesis trials were started by the BrainGate consortium that has been conducting prospective, multicenter North American trials (2004–2008; 2009-ongoing) with implanted Utah arrays connected via a percutaneous connector [49]. From 2004 to 2021, 14 adults with quadriparesis due to spinal cord injury (6 participants), motor neuron disease (6 participants), and brainstem stroke (2 participants) had these systems implanted. The consortium has made significant contributions to clinical neuroscience with research into motor, speech, sleep, and communication via neuroprosthesis, and has progressed engineering aspects such as system development, wireless transmission, and signal processing [12, 14, 50, 51]. A number of other groups use the Utah array platform to conduct implantable neuroprosthetic trials, including the demonstration of brain-controlled functional electrical stimulation to restore anatomical movement [17, 52]. A communication neuroprostheses based on imagined handwriting recognition from recording in the motor hand area has been demonstrated and more recently a high-performance speech neuroprosthesis has been developed by recording directly from the orofacial area of the motor cortex [7, 51].

Despite the apparent infection risk, percutaneous pedestal systems have shown excellent long-term safety. In fourteen participants amassing a collective 17,000 implant days with a system with percutaneous connectors, only a single superficial infection required antibiotics. Over these 17,000 days, not one single participants required hospitalisation, device explantation or intravenous antibiotics for an infection [49]. Nonetheless, a fully implantable system remains the end-goal of many groups working on cortical neuroprosthetics.

**Fig. 5:**
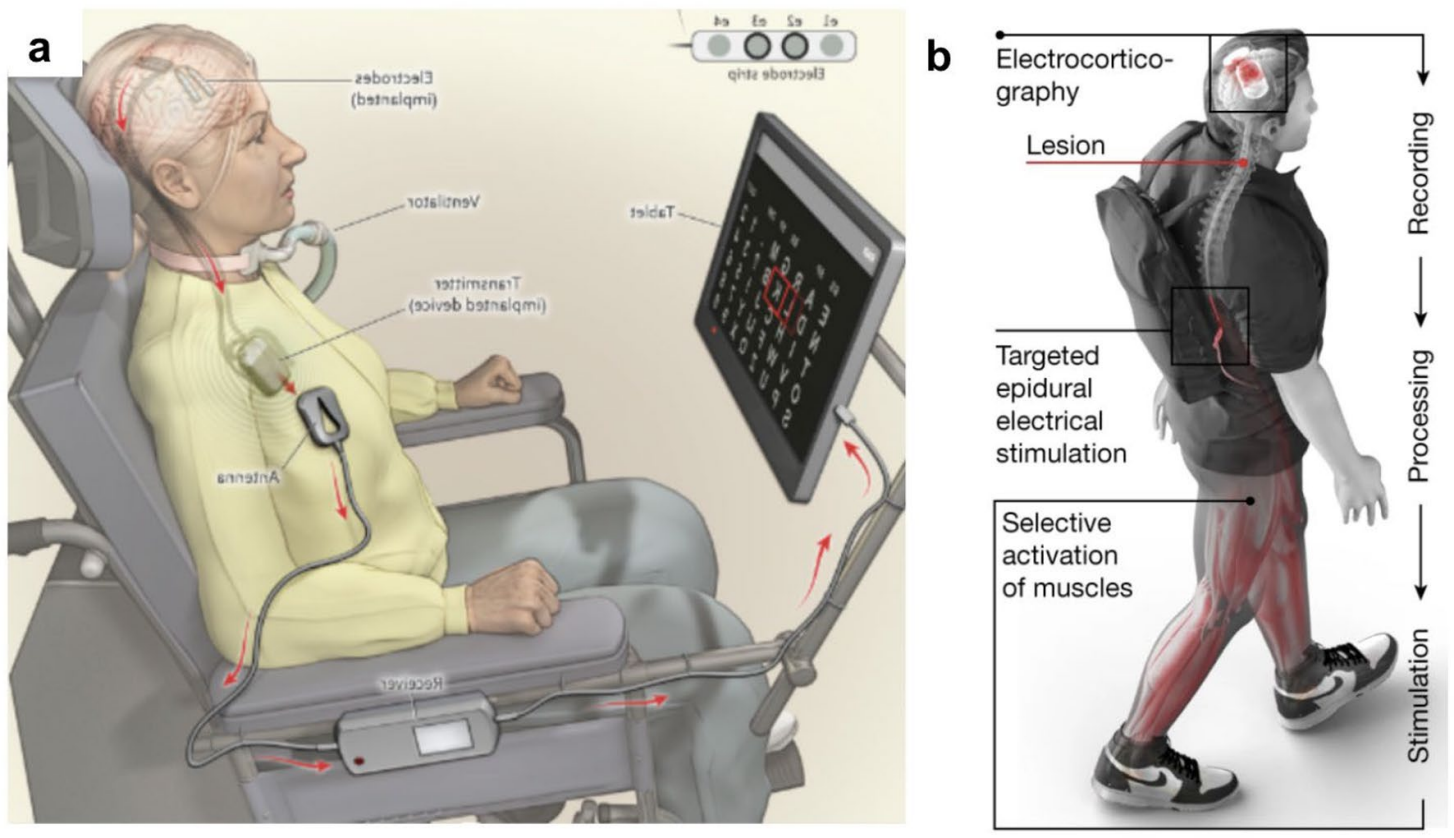
(**a**) The Utrecht motor neuroprosthesis for communication demonstrated in a user with amyotrophic lateral sclerosis (panel adapted from 38 and used with permission). (**b**) The proposed mechanism of the Lausanne Brain Spine Interface for movement restoration demonstrated in a user with spinal cord injury (panel adapted from [10] originally published under Creative Commons Attribution 4.0 International License). Both of these systems are fully implanted and could be used by the participant in their home environment Figure adapted from sources referenced and images reproduced with permission

The first fully implantable cortical neuroprosthetic—the Utrecht neuroprosthesis—was reported in 2016 [38]. This group repurposed existing medical device technology to create a subdural array-based communication neuroprosthesis. Whilst lower performance than the Utah array, this system was remarkable for being fully implantable with no percutaneous connectors and a clinically useful system which the participant was able to rely upon as her primary form of communication as her ALS progressed (Fig. 5a).

**Fig. 6:**
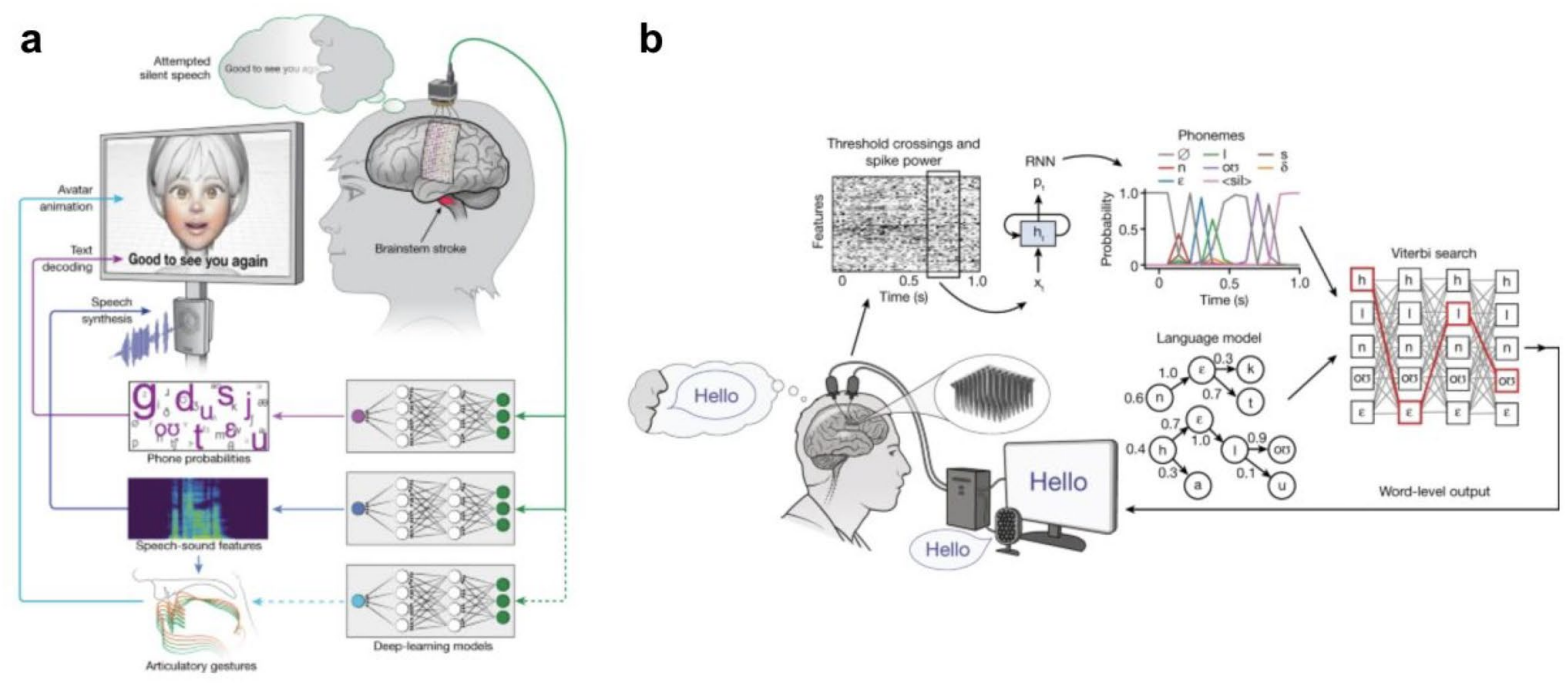
(**a**) The subdural based speech neuroprosthetic with avatar developed in the UCSF BRAVO study9 (panel figure adapted from [9] and used with permission) (**b**) The MEA based speech neuroprosthetic developed as part of the BrainGate consortium (panel figure adapted from [7] originally published under Creative Commons Attribution 4.0 International License). Both these decoders take advantage of language models to improve their accuracy Figure adapted from sources referenced and images reproduced with permission

A percutaneously connected subdural system for communication was subsequently developed in the BCI Restoration of Arm and Voice (BRAVO) trial led by the University of California, San Francisco [39]. This system combined recording from the speech areas of the sensorimotor cortex with a natural language model to generate speech at a rate of 15 words per minute in a participant with brainstem stroke, significantly faster than the Utrecht neuroprosthesis. A second participant in the study was implanted with a higher channel count subdural electrode array (253 channels compared to 128 in the first participant). This higher channel count system was able to achieve a median rate of 78 words per minute with the voice embodied in a virtual avatar which used orofacial movements to convey non-speech communicative gestures. Subdural ECoG recordings suffer less from the nonstationarities described in MEA-based systems, and consequently, both the Utrecht neuroprosthesis and the BRAVO system required minimal recalibration, an important factor in developing a device that is suitable for home use (Fig. 6a).

A recently developed and unique approach to neuroprosthesis development is the endovascular device *Stentrode*. The Stentrode is deployed into the sagittal sinus using standard neurointerventional techniques under angiographic control [11]. The initial clinical trial focussed on participants with ALS to integrate the Stentrode output with eye gaze software to control a tablet computer. Like the Utrecht neuroprosthesis, the Stentrode system has the advantage of being fully implantable.

Whilst many recent developments in neuroprosthetics have focussed on communication or upper extremity mobility devices, in 2023, a group in Lausanne reported their experience with restoration of anatomical movement using a “*Brain-Spine Interface”* [10]. The group combined an extradural recording device, the Wireless Implantable Multi-channel Acquisition system for Generic Interface with Neurons (WIMAGINE) implant developed in Grenoble [19, 53], with an epidural spinal cord stimulator laid over the theca at the level of the twelth thoracic vertebra. Using the neural signals recorded from the WIMAGINE implant, the group was able to modulate specific groups of motor neurons in line with this intention and thus restoring some muscle and joint movement. In this application, as well as an improvement in lower limb power with the device, the participant also gained a neurorehabilitative benefit which was sustained even without the device active (Fig. 5b).

Although not yet reported in the scientific literature, Neuralink Corporation implanted their N1 cortical implant in January 2024 in a patient with quadriplegia. The N1 system is based upon 1,024 electrodes across 64 flexible ‘threads’ which are robotically implanted into the motor cortex, connected to a small implanted electronics transmitter that wirelessly sends the signal to the external data processor. Shortly after this first implantation, Neuralink announced that some of the threads had retracted from the participant’s brain, reducing the number of recording sites. According to information released by the company, the study participant is able to continue using the device both for study tasks and independent use even after this reduction in the number of usable electrodes [54].

In addition to these devices which have been implanted, there are a number of new devices and approaches in development by industry which are working towards their first clinical trials. These include intracortical microelectrode arrays for high bandwidth brain–computer interfaces made by Paradromics in Austin, Texas; the Brain Interchange 1 subdural electrode platform made by Cortec in Freiburg, Germany; and a subdural electrode-based platform delivered by a cranial microslit technique made by Precision Neuroscience in New York. It remains to be seen which of these approaches will ultimately successfully translate into clinical practice but all are expected to move into first in human clinical trials in the coming years.

### Ethical concerns

Implanted cortical neuroprosthetics raise specific ethical concerns in addition to those raised more generally by neuroprosthetics and other neurotechnology. Although not unique to neuroprosthetics, users run a risk of being technologically isolated if they are implanted with devices and then left unsupported due to either insolvency of the manufacturer or the manufacturer taking the view that it is outdated and deciding to no longer support it [55–57].

Security of neural data is an important area of ethical concerns in relation to cortical neuroprosthetics in general, as reflected by the growth of dual-use and misuse issues around neurotechnology [58–60], and the emerging fields of neurosecurity and neurocybersecurity [55]. Medical devices can be susceptible to cyberattacks, but neuroprosthetics are particularly so because their signals are seldom encrypted to prevent augmenting the latency between the signal and its end result (e.g. the movement of a prosthetic limb) and also drawing more power from batteries operating remote devices. Self-hacking to gain additional enhancement from a neuroprosthetic is also identified as raising potential concerns for both security and safety [55].

Informed consent and further, the capacity to consent, is a crucial concern in relation to implanted neuroprosthetics [61]. Assessing capacity can be challenging in users with communication impairments who are one of the first groups who may benefit from these devices. In addition, users need to be fully consented not just for the procedural risks but also the possibility of their device being no longer supported and requiring explantation. A concern that has already been raised about DBS devices is that stimulation with these implants may affect users’ personality and judgement [62]. As neuroprosthetics become more sophisticated, this may become an issue for this class of devices also, raising the issue of whether users’ decision making capacity can be compromised by the device itself [61, 62].

As neuroprosthetic technology becomes more mature, it will likely benefit wider groups of users, with less severe impairments than is currently the case. This entails the potential for initial therapeutic goals moving towards non-clinical or “enhancement” applications [63, 64].

Whilst clearly someway away from the current generation of devices, it has been suggested that cortical neuroprosthetics could have transhumanist implications in the future. In particular, that such devices may not just be adopted by able bodied people, but that they may become a central part of how humans interact with the metaverse and artificial intelligence systems.

The use of devices from non-medical applications also raises questions of equity, with a risk of disenfranchising those segments of the population unable to access “enhancing” neurotechnologies [65].

In the case when speech neuroprosthetics are used to support high-stakes communications, listeners must trust that they can evaluate the accuracy, intent and voluntariness of what is communicated. The technology design should incorporate the roles and needs of listeners [66]. More generally, there is a serious need for studies of carers’ perspectives, roles and needs in relation to neuroprosthetics [67], as well as of users’ perspectives [66, 68]. This points at the necessity to involve empirical, qualitative social scientists in clinical trials and early clinical deployments to help understand the full impact of these novel devices on users and their carers.

### Which groups will be first to benefit from implanted cortical neuroprosthetics?

There is a clinical need for technologies which can restore the ability of people with paralysis to communicate with others as well as interact with the digital environment which is increasingly interwoven into everyday life. In a survey of patients with locked-in syndrome communication, computer use and environmental control were found to be the most desired neuroprosthetic applications [69].

People who are locked-in secondary to brainstem stroke or ALS are often thought to have amongst the best indications for communication restoration and have been the focus of recent high profile successes with the USCF Speech Neuroprosthesis [9] and the Stentrode, respectively [15]. A challenge for surgical implantation in this group is that the process causing their communication impairment frequently co-associates with severe bulbar dysfunction or respiratory compromise, increasing their risk for general anaesthesia. In the specific case of ALS, there are further risks associated with the use of muscle relaxants that increase the risk of general anaesthesia [70].

Many people with severe communication impairment rely on augmentative and alternative communication (AAC) strategies such as gaze control interfaces. The Stentrode explicitly leverages this by augmenting existing gaze control technology with a switch from the implant itself. A group that presently make up a large number of users of gaze control devices are adults and children with cerebral palsy [71]. They are presently under-represented in communication trials but may benefit as communication neuroprosthetics move into the mainstream.

People with spinal cord injury, multiple sclerosis, brainstem stroke, muscular dystrophies, and amputations have been candidates for trials aiming at movement restoration. The recent demonstration of a brain–spine interface using the relatively stable signal provided by an extradural ECoG array is an example of a physical movement restoring neuroprosthetic which the user was able to use in their own home with a sustained clinical benefit [10]. This is a paradigm by which ICNs for physical movement restoration may first translated into routine clinical use.

## Conclusions and future directions

Almost two decades after the first BrainGate trial, implanted cortical neuroprosthetics are on the cusp of translation into clinically useful devices with the prospect of real participant benefit being demonstrated in clinical trials [15]. Over the coming decades, we anticipate implanted cortical neuroprosthetics will first enter mainstream clinical practice in two contexts. First, for recovery of anatomical movement in people with paralysis where stimulation of the user’s native musculoskeletal system will contribute both to direct restoration of movement as well as long-term rehabilitation. Second, for communication restoration for tetraplegic users where they may translate thought to speech or give control over a tablet computer giving these people a new gateway to express their thoughts and control their environment. An essential part of this clinical translation will be ensuring that these devices are reliable, provide stable signals over the device lifetime and always available for people to use in their everyday lives and own homes. Advances in device hardware (e.g. higher bandwidth and higher neuron count recordings) and software (e.g. decoders, compression algorithms, communication protocols) will further increase the performance of neuroprosthetics and expand their capabilities.

As ICNs become more sophisticated, it is likely that they will begin to provide benefit to an even wider cohort of people with less severe impairments than those that have been considered for these initial trials.

## Search strategy and selection criteria

The initial source for the literature referenced in this review was identified through a systematic scoping review of the Ovid MEDLINE and Embase databases. The search strategy included a combination of keywords and MeSH terms related to “brain-computer interface,” and “prosthesis”. There was no limit to the start date but the systematic search was completed on August 31st 2022. This was supplemented by further searching of the literature up until submission of this review to ensure to it was up to date in this rapidly changing field. Additional papers were identified from the authors’ knowledge of the literature and from reference lists of identified publications.

We included studies that met the following criteria: primary research articles reporting on the development, application, or evaluation of neuroprosthetic BCIs; studies involving users with evidence of device implantation; and studies that assessed clinical outcomes, safety, or feasibility of neuroprosthetic BCI use.

## Data Availability

Not applicable.

## Abbreviations

AAC: Augmentative and alternative communication
ALS: Amyotrophic lateral sclerosis
BCI: Brain–computer interface
BSI: Brain–spine interface
BRAVO: BCI restoration of arm and voice
DBS: Deep brain stimulation
EEG: Electroencephalogram
ECoG: Electrocorticography
FES: Functional electrical stimulation
MEA: Microelectrode array
MRI: Magnetic resonance imaging
SCI: Spinal cord injury
STIMO-BSI: Brain-controlled spinal cord stimulation in patients with spinal cord injury
WIMAGINE: Wireless implantable multi-channel acquisition system for generic interface with neurons

## Glossary

Biocompatibility: The ability of a material to safely interact with the host and sustain its intended function in a biological context.
BrainGate: A consortium of investigators who conducted the first multicentre implanted cortical neuroprosthesis trials with intracranial microelectrode arrays.
BCI restoration of arm and voice (BRAVO): A clinical trial, led by the University of California, San Francisco that combined recording from speech areas of the sensorimotor cortex with a natural language model to synthesise speech.
Calibration: The process of iteratively modifying a decoding algorithm to correlate neural signals and intention.
Decoder: A mathematical algorithm that converts neural data into a “command signal” that is used to drive an effector.
Electrocorticography (ECoG): The technique of measuring neural signals through an array of electrodes placed on the surface of the brain.
Implanted/intracranial cortical neuroprosthetic (ICN): A novel class of medical devices developed to replace dysfunctional neural pathways by facilitating direct information exchange between the cerebral cortex and a digital system which can facilitate interaction with the external world.
Microelectrode array (MEA): A device containing multiple individual recording electrodes. In the context of implantable cortical neuroprosthetics, the most common MEA is the ‘Utah Array’ which is typically a 10x10 array (i.e. 100 individual shanks).
Neurotechnology: A range of techniques and devices which interact with the nervous system to record or modulate neural activity.
Single unit: A recorded electrical signal consistent with the discharge of an individual neuron.
Stentrode: A neuroprosthetic developed in Utrecht for facilitating communication based on a subdural array.
Utrecht neuroprosthesis: A neuroprosthetic developed in Utrecht for facilitating communication based on a subdural array.
Wireless implantable multi-channel acquisition system for generic interface with neurons (WIMAGINE): A wireless epidural electrocorticography neuroprosthetic.

## References

[1] Daly JJ, Wolpaw JR (2008) Brain-computer interfaces in neurological rehabilitation. Lancet Neurol 7:1032–1043 18835541 10.1016/S1474-4422(08)70223-0

[2] An interface connects. *Nat. Electron.* **6**, 89–89 (2023)

[3] Pfurtscheller, G. *et al.* Graz-BCI: state of the art and clinical applications. *IEEE Trans. Neural Syst. Rehabil. Eng. Publ. IEEE Eng. Med. Biol. Soc.* **11**, 177–180 (2003). 10.1109/TNSRE.2003.814454 12899267

[4] Lenarz T, Büchner A, Illg A (2022) Cochlear implantation: concept, results outcomes and quality of life. Laryngorhinootologie 101:S36–S78 35605612 10.1055/a-1731-9321

[5] Benabid AL et al (2011) Deep brain stimulation: BCI at large, where are we going to? Prog Brain Res 194:71–82 21867795 10.1016/B978-0-444-53815-4.00016-9

[6] Zhao Z-P et al (2023) Modulating brain activity with invasive brain–computer interface: a narrative review. Brain Sci 13:134 36672115 10.3390/brainsci13010134 PMC9856340

[7] Willett FR et al (2023) A high-performance speech neuroprosthesis. Nature 620:1031–1036 37612500 10.1038/s41586-023-06377-x PMC10468393

[8] Caspi A et al (2021) Eye movements and the perceived location of phosphenes generated by intracranial primary visual cortex stimulation in the blind. Brain Stimulat 14:851–860 10.1016/j.brs.2021.04.019 33991713

[9] Metzger SL et al (2023) A high-performance neuroprosthesis for speech decoding and avatar control. Nature 620:1037–1046 37612505 10.1038/s41586-023-06443-4 PMC10826467

[10] Lorach H et al (2023) Walking naturally after spinal cord injury using a brain-spine interface. Nature. 10.1038/s41586-023-06094-5 37225984 10.1038/s41586-023-06094-5 PMC10232367

[11] Mitchell P et al (2023) Assessment of safety of a fully implanted endovascular brain-computer interface for severe paralysis in 4 patients: the stentrode with thought-controlled digital switch (SWITCH) study. JAMA Neurol 80:270–278 36622685 10.1001/jamaneurol.2022.4847 PMC9857731

[12] Nuyujukian P et al (2018) Cortical control of a tablet computer by people with paralysis. PLoS ONE 13:e0204566 30462658 10.1371/journal.pone.0204566 PMC6248919

[13] Ramsey NF, Crone NE (2023) Brain implants that enable speech pass performance milestones. Nature 620:954–955 37612488 10.1038/d41586-023-02546-0

[14] Hochberg LR et al (2006) Neuronal ensemble control of prosthetic devices by a human with tetraplegia. Nature 442:164–171 16838014 10.1038/nature04970

[15] Oxley TJ et al (2021) Motor neuroprosthesis implanted with neurointerventional surgery improves capacity for activities of daily living tasks in severe paralysis: first in-human experience. J NeuroInterventional Surg 13:102–108 10.1136/neurintsurg-2020-016862 PMC7848062 33115813

[16] Sawyer A, Cooke L, Ramsey NF, Putrino D (2023) The digital motor output: a conceptual framework for a meaningful clinical performance metric for a motor neuroprosthesis. J NeuroInterventional Surg. 10.1136/jnis-2023-020316 10.1136/jnis-2023-020316 37524520

[17] Ajiboye AB et al (2017) Restoration of reaching and grasping movements through brain-controlled muscle stimulation in a person with tetraplegia: a proof-of-concept demonstration. Lancet 389:1821–1830 28363483 10.1016/S0140-6736(17)30601-3 PMC5516547

[18] Zhang Y, Liu X, Qiao X, Fan Y (2023) Characteristics and emerging trends in research on rehabilitation robots from 2001 to 2020: bibliometric study. J Med Internet Res 25:e42901 37256670 10.2196/42901 PMC10267796

[19] Benabid AL et al (2019) An exoskeleton controlled by an epidural wireless brain-machine interface in a tetraplegic patient: a proof-of-concept demonstration. Lancet Neurol 18:1112–1122 31587955 10.1016/S1474-4422(19)30321-7

[20] Vilela M, Hochberg LR (2020) Chapter 8—Applications of brain-computer interfaces to the control of robotic and prosthetic arms. In: Ramsey NF, Millán JR (eds) Handbook of clinical neurology, vol 168. Elsevier, Amsterdam, pp 87–99 10.1016/B978-0-444-63934-9.00008-1 32164870

[21] Young MJ, Lin DJ, Hochberg LR (2021) Brain-computer interfaces in neurorecovery and neurorehabilitation. Semin Neurol 41:206–216 33742433 10.1055/s-0041-1725137 PMC8768507

[22] van Dokkum LEH, Ward T, Laffont I (2015) Brain computer interfaces for neurorehabilitation—its current status as a rehabilitation strategy post-stroke. Ann Phys Rehabil Med 58:3–8 25614021 10.1016/j.rehab.2014.09.016

[23] Jarosiewicz B et al (2015) Virtual typing by people with tetraplegia using a self-calibrating intracortical brain-computer interface. Sci Transl Med 7:313179 10.1126/scitranslmed.aac7328 PMC4765319 26560357

[24] Thielen B, Meng E (2021) A comparison of insertion methods for surgical placement of penetrating neural interfaces. J Neural Eng 18:041003. 10.1088/1741-2552/abf6f2 10.1088/1741-2552/abf6f2 PMC8600966 33845469

[25] Musk E (2019) An integrated brain-machine interface platform with thousands of channels. J Med Internet Res 21:e16194 31642810 10.2196/16194 PMC6914248

[26] Neuralink. PRIME Study Progress Update — User Experience. *Neuralink Blog* https://neuralink.com/blog/prime-study-progress-update-user-experience/ (2024).

[27] Mestais CS et al (2015) WIMAGINE: wireless 64-channel ECoG recording implant for long term clinical applications. IEEE Trans Neural Syst Rehabil Eng 23:10–21 25014960 10.1109/TNSRE.2014.2333541

[28] The Technology. *Synchron* https://synchron.com/technology

[29] Epilepsy | Ad-Tech Medical. https://adtechmedical.com/epilepsy

[30] Wang W et al (2009) Human motor cortical activity recorded with micro-ECoG electrodes during individual finger movements. Conf. Proc. Annu. Int. Conf. IEEE Eng. Med. Biol. Soc. IEEE Eng. Med. Biol. Conf. 2009:586–589 10.1109/IEMBS.2009.5333704 PMC3142578 19964229

[31] Precision - Product. https://precisionneuro.io/product.

[32] Larzabal C et al (2021) Long-term stability of the chronic epidural wireless recorder WIMAGINE in tetraplegic patients. J Neural Eng 18:056026 10.1088/1741-2552/ac2003 34425566

[33] Labeyrie M-A et al (2021) Intracranial venous sinus stenting for the treatment of lateral sinus stenoses: an analysis of 200 patients. Diagn Interv Imaging 102:619–627 34127434 10.1016/j.diii.2021.05.008

[34] Truccolo W, Friehs GM, Donoghue JP, Hochberg LR (2008) Primary motor cortex tuning to intended movement kinematics in humans with tetraplegia. J Neurosci 28:1163 18234894 10.1523/JNEUROSCI.4415-07.2008 PMC6671402

[35] Schoonover CE, Ohashi SN, Axel R, Fink AJP (2021) Representational drift in primary olfactory cortex. Nature 594:541–546 34108681 10.1038/s41586-021-03628-7

[36] Fan C et al (2023) Plug-and-play stability for intracortical brain-computer interfaces: a one-year demonstration of seamless brain-to-text communication. Adv Neural Inf Process Syst 36:42258–42270 38738213 PMC11086983

[37] Crone NE, Sinai A, Korzeniewska A (2006) High-frequency gamma oscillations and human brain mapping with electrocorticography. Prog Brain Res 159:275–295 17071238 10.1016/S0079-6123(06)59019-3

[38] Vansteensel MJ et al (2016)cain-computer interface in a locked-in patient with ALS. N Engl J Med 375:2060–2066 27959736 10.1056/NEJMoa1608085 PMC5326682

[39] Moses DA et al (2021) Neuroprosthesis for decoding speech in a paralyzed person with anarthria. N Engl J Med 385:217–227 34260835 10.1056/NEJMoa2027540 PMC8972947

[40] Woeppel K et al (2021) Explant analysis of utah electrode arrays implanted in human cortex for brain-computer-interfaces. Front Bioeng Biotechnol. 10.3389/fbioe.2021.759711 34950640 10.3389/fbioe.2021.759711 PMC8688945

[41] Sponheim C et al (2021) Longevity and reliability of chronic unit recordings using the Utah, intracortical multi-electrode arrays. J Neural Eng 18:066044 10.1088/1741-2552/ac3eaf PMC8981395 34847547

[42] Patel PR et al (2023) Utah array characterization and histological analysis of a multi-year implant in non-human primate motor and sensory cortices. J Neural Eng 20:014001 10.1088/1741-2552/acab86 PMC9954796 36595323

[43] Cody PA, Eles JR, Lagenaur CF, Kozai TDY, Cui XT (2018) Unique electrophysiological and impedance signatures between encapsulation types: an analysis of biological Utah array failure and benefit of a biomimetic coating in a rat model. Biomaterials 161:117–128 29421549 10.1016/j.biomaterials.2018.01.025 PMC5817007

[44] He F, Lycke R, Ganji M, Xie C, Luan L (2020) Ultraflexible neural electrodes for long-lasting intracortical recording. iScience 23:101387 32745989 10.1016/j.isci.2020.101387 PMC7398974

[45] Tang X, Shen H, Zhao S, Li N, Liu J (2023) Flexible brain–computer interfaces. Nat Electron 6:109–118

[46] Marblestone A et al (2013) Physical principles for scalable neural recording. Front Comput Neurosci. 10.3389/fncom.2013.00137 24187539 10.3389/fncom.2013.00137 PMC3807567

[47] Diaz RE, Sebastian T (2013) Electromagnetic limits to radiofrequency (RF) neuronal telemetry. Sci Rep 3:3535 24346503 10.1038/srep03535 PMC3866607

[48] Kennedy PR, Bakay RAE (1998) Restoration of neural output from a paralyzed patient by a direct brain connection. NeuroReport 9:1707 9665587 10.1097/00001756-199806010-00007

[49] Rubin DB et al (2023) Interim safety profile from the feasibility study of the BrainGate neural interface system. Neurology 100:e1177–e1192 36639237 10.1212/WNL.0000000000201707 PMC10074470

[50] Brandman DM et al (2018) Rapid calibration of an intracortical brain-computer interface for people with tetraplegia. J Neural Eng 15:026007 29363625 10.1088/1741-2552/aa9ee7 PMC5823702

[51] Willett FR, Avansino DT, Hochberg LR, Henderson JM, Shenoy KV (2021) High-performance brain-to-text communication via handwriting. Nature 593:249–254 33981047 10.1038/s41586-021-03506-2 PMC8163299

[52] Bouton CE et al (2016) Restoring cortical control of functional movement in a human with quadriplegia. Nature 533:247–250 27074513 10.1038/nature17435

[53] Moly A et al (2022) An adaptive closed-loop ECoG decoder for long-term and stable bimanual control of an exoskeleton by a tetraplegic. J. Neural Eng. 19:026021 10.1088/1741-2552/ac59a0 35234665

[54] Neuralink. PRIME Study Progress Update. *Neuralink Blog* https://neuralink.com/blog/prime-study-progress-update/ (2024).

[55] Liv N, Greenbaum D (2023) Cyberneurosecurity. In: Dubljević V, Coin A (eds) Policy, identity, and neurotechnology: the neuroethics of brain-computer interfaces. Springer International Publishing, Cham, pp 233–251. 10.1007/978-3-031-26801-4_13

[56] Cabrera LY, Weber DJ (2023) Rethinking the ethical priorities for brain–computer interfaces. Nat Electron 6:99–101

[57] Drew L (2022) Abandoned: the human cost of neurotechnology failure. Nature. 10.1038/d41586-022-03810-5 36474050 10.1038/d41586-022-03810-5

[58] Butorac I, Lentzos F, Aicardi C (2021) Gray matters: exploring technologists’ perceptions of dual-use potentiality in emerging neurotechnology applications. Health Secur 19:424–430 34264762 10.1089/hs.2020.0147

[59] Tara Mahfoud, Christine Aicardi, Saheli Datta, & Nikolas Rose. The Limits of Dual Use. *Issues in Science and Technology* https://issues.org/the-limits-of-dual-use/ (2018).

[60] Aicardi C et al (2018) Opinion on ‘responsible dual use’ political, security. Intell Milit Res Concern Neurosci Neurotechnol. 10.5281/zenodo.4588601

[61] Tubig P, Gilbert F (2023) “The trauma of losing your own identity again”: the ethics of explantation of brain-computer interfaces. In: Dubljević V, Coin A (eds) Policy, identity, and neurotechnology: the neuroethics of brain-computer interfaces. Springer International Publishing, Cham, pp 27–41. 10.1007/978-3-031-26801-4_3

[62] Wilt JA, Merner AR, Zeigler J, Montpetite M, Kubu CS (2021) Does personality change follow deep brain stimulation in Parkinson’s disease patients? Front Psychol. 10.3389/fpsyg.2021.643277 34393883 10.3389/fpsyg.2021.643277 PMC8361492

[63] Wolbring G (2008) The politics of ableism. Development 51:252–258

[64] Wolbring, G. Ability Privilege: A Needed Addition to Privilege Studies. SSRN Scholarly Paper at https://papers.ssrn.com/abstract=2487616 (2014).

[65] Wolbring G (2021) Auditing the impact of neuro-advancements on health equity. J Neurol Res 12:54–68

[66] Soekadar SR et al (2023) Future developments in brain/neural–computer interface technology. In: Dubljević V, Coin A (eds) Policy, identity, and neurotechnology: the neuroethics of brain-computer interfaces. Springer International Publishing, Cham, pp 65–85

[67] Kögel J, Schmid JR, Jox RJ, Friedrich O (2019) Using brain-computer interfaces: a scoping review of studies employing social research methods. BMC Med Ethics 20:18 30845952 10.1186/s12910-019-0354-1 PMC6407281

[68] Feinsinger A et al (2022) Ethical commitments, principles, and practices guiding intracranial neuroscientific research in humans. Neuron 110:188–194 35051364 10.1016/j.neuron.2021.11.011 PMC9417025

[69] Branco MP et al (2021) Brain-computer interfaces for communication: preferences of individuals with locked-in syndrome. Neurorehabil Neural Repair 35:267–279 33530868 10.1177/1545968321989331 PMC7934157

[70] Kim HT et al (2022) Total intravenous anesthesia without muscle relaxant for pulmonary wedge resection in a patient with amyotrophic lateral sclerosis: a case report. Am J Transl Res 14:3554–3558 35702109 PMC9185035

[71] Hemmingsson H, Borgestig M (2020) Usability of eye-gaze controlled computers in Sweden: a total population survey. Int J Environ Res Public Health 17:1639 32138358 10.3390/ijerph17051639 PMC7084643

